# A modified VMAT adaptive radiotherapy for nasopharyngeal cancer patients based on CT-CT image fusion

**DOI:** 10.1186/1748-717X-8-277

**Published:** 2013-11-27

**Authors:** Xiance Jin, Ce Han, Yongqiang Zhou, Jinling Yi, Huawei Yan, Congying Xie

**Affiliations:** 1 Radiotherapy and Chemotherapy, Department of the 1st Affiliated Hospital of Wenzhou Medical University, No.2 Fuxue Lane, Wenzhou 325000, China

**Keywords:** Nasopharyngeal cancer, Adaptive radiotherapy, Volumetric modulated arc therapy, CT-CT image fusion, Deformable registration

## Abstract

**Background:**

To investigate the feasibility and benefits of a modified adaptive radiotherapy (ART) by replanning in the initial CT (iCT) with new contours from a repeat CT (rCT) based on CT-CT image fusion for nasopharyngeal cancer (NPC) patients underwent volumetric modulated arc radiotherapy (VMAT).

**Materials and methods:**

Nine NPC patients underwent VMAT treatment with a rCT at 23rd fraction were enrolled in this study. Dosimetric differences for replanning VMAT plans in the iCT and in the rCT were compared. Volumetric and dosimetric changes of gross tumor volume (GTV) and organs at risk (OARs) of this modified ART were also investigated.

**Results:**

No dosimetric differences between replanning in the iCT and in the rCT were observed. The average volume of GTV decreased from 78.83 ± 38.42 cm^3^ in the iCT to 71.44 ± 37.46 cm^3^ in the rCT, but with no significant difference (p = 0.42).The average volume of the left and right parotid decreased from 19.91 ± 4.89 cm^3^ and 21.58 ± 6.16 cm^3^ in the iCT to 11.80 ± 2.79 cm^3^ and 13.29 ± 4.17 cm^3^ in the rCT (both p < 0.01), respectively. The volume of other OARs did not shrink very much. No significant differences on PTV_GTV_ and PTV_CTV_ coverage were observed for replanning with this modified ART. Compared to the initial plans, the average mean dose of the left and right parotid after re-optimization were decreased by 62.5 cGy (p = 0.05) and 67.3 cGy (p = 0.02), respectively, and the V5 (the volume receiving 5 Gy) of the left and right parotids were decreased by 7.8% (p = 0.01) and 11.2% (p = 0.001), respectively. There was no significant difference on the dose delivered to other OARs.

**Conclusion:**

Patients with NPC undergoing VMAT have significant anatomic and dosimetric changes to parotids. Repeat CT as an anatomic changes reference and re-optimization in the iCT based on CT-CT image fusion was accurate enough to identify the volume changes and to ensure safe dose to parotids.

## Introduction

Due to its dose painting capability and sharp dose gradient, intensity modulated radiotherapy (IMRT), and recently developed new IMRT delivery method: volumetric modulated arc therapy (VMAT), have been accepted as the primary treatment modalities for nasopharyngeal cancer (NPC) patients [1,2]. Studies have confirmed that the dosimetric advantages of IMRT over conventional treatment translated into clinical outcome with reduced parotid toxicity [3]. However, geometry and anatomic changes during the long course of IMRT treatment have limited the clinical benefits of IMRT [4]. Adaptive radiotherapy (ART) is a formal approach to correct for daily tumor and normal tissue variations through online or offline modification of original IMRT target volumes and plans [5]. In most of the clinical workflows, the adaption of the plan occurs in a replanning computed tomography (CT) on the basis of relevant dosimetric discrepancies.

One retrospective study demonstrated that repeat CT (rCT) imaging and IMRT replanning helped to ensure adequate doses to target volumes and safe doses to normal structures for patients who had clinically identified anatomic changes during the course of IMRT [6]. It had been reported that NPC patients can benefit from replaning before the 25th fraction [7]. The 3-year local progression-free survival of advanced NPC patients had also been improved by IMRT replanning [8]. One potential problem of replanning IMRT with a second CT scan is the loss of accurate dose accumulation on organs at risk (OARs) and targets due to several potential discrepancies between the iCT and the rCT. Hybrid IMRT plan by applying the beam configurations of the first IMRT plan to the anatomy of the second CT scan was usually applied to study the accumulated dose on OARs and target volumes [6-8]. However, the volume of targets and OARs for hybrid IMRT plan were different from those in the iCT due to anatomic changes and inherent delineation variations [9,10]. It is hard for physicians to delineate the same superior and inferior boundary of targets in two different CT sets. It might also introduce additional errors during the transmission of the beam configuration due to the beam isocenter displacement resulted from volume changes. Therefore, the accumulated dose with hybrid IMRT in the rCT could be very different from that in the initial planning CT, caused by above mentioned discrepancies and the feature difference between two CT image sets.

In a previous dosimetric evaluation study of a three-phase ART for NPC, Fung et al. generated 2 hybrid plans using original contours pasted on the rCTs based on CT-CT image fusion to study the accumulate dose [11]. We hypothesize that it is also reasonable to adapt the ART for NPC patients by relying on the iCT for replanning with new contours projected from a rCT based on CT-CT image fusion. A composite plan could be easily generated on one image set with two prescriptions, therefore, accumulation dose could be accurately calculated, especially the accumulation dose to brainstem and spinal cord could be accurately constrained within their tolerant dose.

The purpose of this study is to study the feasibility of this modified ART based on CT-CT image fusion, and to investigate the volumetric change and dosimetric benefits of this modified ART.

## Materials and methods

### Study design

**Figure 1:**
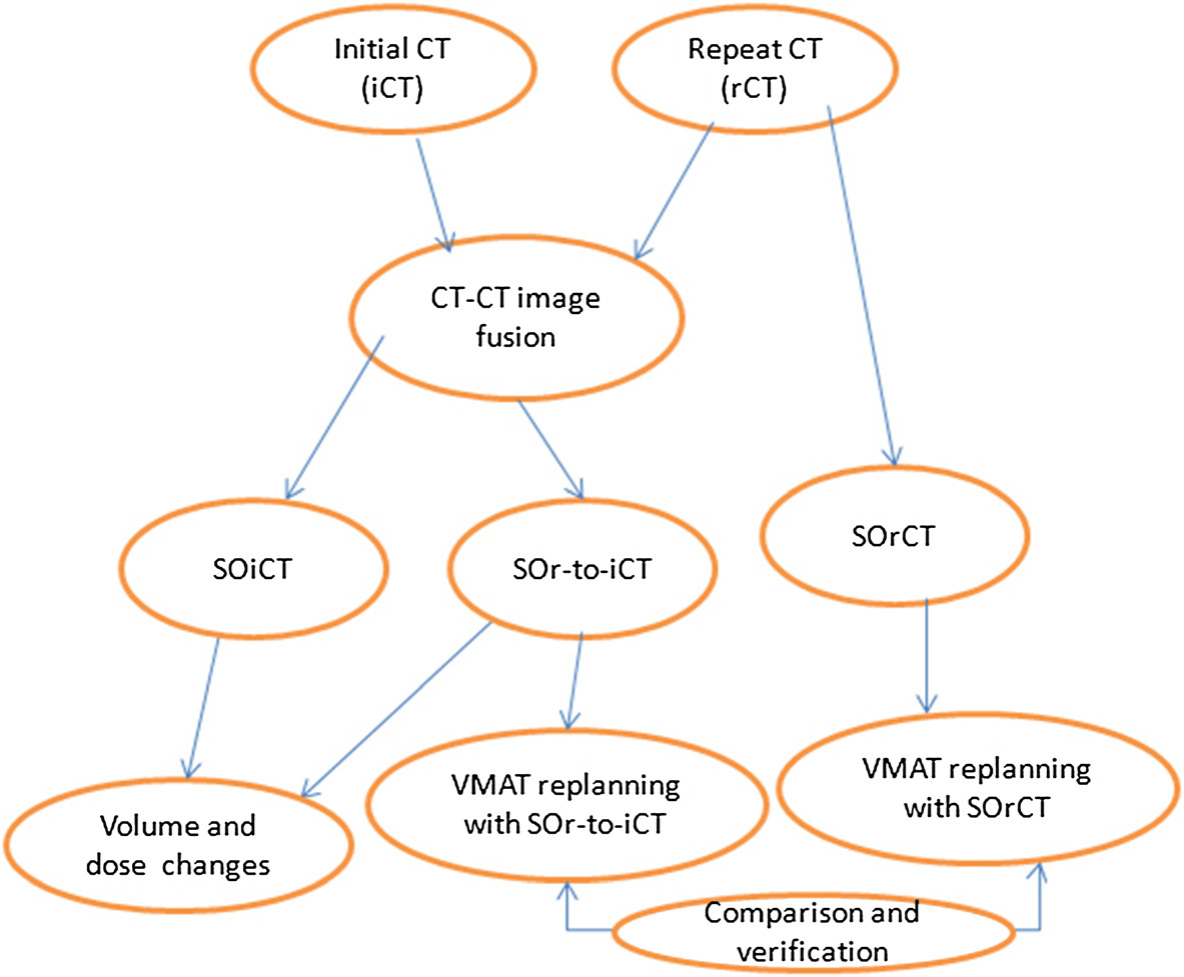
**Flowchart for the whole study design.** iCT is the initial CT; rCT is the repeat CT; SOiCT is the structures on the initial CT; SOrCT is the structures on the repeat CT; SOr-to-iCT is the structures propagated from the repeat CT on the initial CT.

As shown in Figure 1 the flowchart for the overall study design, two image sets iCT and rCT with structures on the iCT (SOiCT) and rCT (SOrCT) were firstly fused based on CT-CT image fusion. With the propagation of SOrCT on the iCT, additional set of structures on iCT were generated (SOr-to-iCT). Dosimetric differences between VMAT replanning with SOr-to-iCT and VMAT replanning with SOrCT were analyzed to study and feasibility of replanning with iCT for adaptive VMAT. Volumetric variations and resulted dosimetric effects were also investigated to study the volume change effects during VMAT.

### Patients

This study was approved by the Institutional Review Board and performed at the 1st Affiliated Hospital of Wenzhou Medical University. We retrospectively reviewed 28 NPC patients treated by dual arc VMAT between January 2011 and November 2012. Nine patients had a rCT and replanning at the 23rd fraction during their course of treatment due to observed anatomic changes (including tumor shrinkage, nodal shrinkage and/or weight loss). All the patients had NPC with AJCC stage II-IV, as summarized in Table 1.

**Table 1:** Patients characteristics

**Patients**	**Staging**	**GTV volume (cm**^**3**^**)**	**CTV volume (cm**^**3**^**)**
1	T2N2Mx	59.61	617.99
2	T2N1Mx	85.49	757.70
3	T3N0M0	62.18	589.08
4	T4N0Mx	170.96	546.76
5	T3N3Mx	92.39	504.18
6	T2N2Mx	64.38	416.45
7	T4N0Mx	83.41	739.74
8	T2N2Mx	45.27	498.05
9	T2N3Mx	45.79	462.43

### Treatment planning

Before treatment, all patients underwent immobilization with a thermoplastic head-and-shoulder mask in the supine position. CT simulation was acquired on a Philips Brilliant spiral CT (Philips Brilliant, Cleveland, OH, USA) according to standard procedures with 3 mm slice spacing. Magnetic resonance images were fused into simulation CT images to assist the target delineation.

Targets and OAR contours had been reported in our previous study and summarized here [12]. Gross tumor volume (GTV) was defined as the mass shown in the enhanced CT images, including the nasopharyngeal tumor, retropharyngeal lymphadenopathy, and enlarged neck nodes. Clinical target volume (CTV) was usually defined as the GTV plus a margin of potential microscopic spread, including the nasopharynx, retropharyngeal nodes, clivus, skull base, pterygoid fossae, parapharyngeal space, inferior sphenoid sinus, posterior third of the nasal cavity, and maxillary sinuses. Planning target volume (PTV) was created based on target volume plus 3 mm margin, allowing for setup variability.

The prescription doses to the planning target volume of GTV (PTV_GTV_) and CTV (PTV_CTV_) were 2.5 Gy and 2.0 Gy per fraction, respectively. Total of 28 fractions were prescribed with a total dose of 70 Gy and 56 Gy for PTV_GTV_ and PTV_CTV_, respectively. OARs of brainstem, spinal cord, mandible, left and right parotid were constrained for optimization. Dual arc VMAT plans were optimized with the SmartArc algorithm in Pinnacle treatment planning system (TPS) (Philips, Fichburg, WI, USA). VMAT objective settings and optimization parameters has been reported in our previous study [13].

### CT re-scanning and VMAT replanning

The rCT was acquired at the 23rd fraction for these patients due to clinically observed changes in patients’ anatomy (by inspection, palpation, and /or direct endoscopy). Identical patient position and orientation were maintained for two CT scans. The second CT was fused into the iCT with rigid CT-CT image fusion based on bony landmarks in Pinnacle TPS. CTV was firstly transferred from the iCT into the rCT using a propagation tool from Pinnacle^3^ to check the volumetric change of this high risk volume. No dramatic changes on CTV contours were necessary based on the physician’s decision for these patients. GTV and OARs in the rCT were manually contoured and transferred to the iCT with the propagation tool. For the modified ART, a replanning VMAT plan was optimized in the iCT based on the SOr-to-iCT. The prescription doses for PTV_GTV_ and PTV_CTV_ for the remaining 5-fraction replanning VMAT plans were 12.5 Gy and 10 Gy, respectively.

Another replanning VMAT plan in the rCT was also optimized with identical optimization parameters and objective settings. Dosimetric differences between these two group replanning VMAT plans were compared to study the feasibility of this modified ART based on CT-CT image fusion.

The volume changes of GTV and OARs for this modified ART between the iCT and the rCT, as well as the dosimetric effects of volumetric changes, were also investigated. The dose distribution of the initial plans for the remaining 5 fractions were projected on SOr-to-iCT without reoptimization to study the dosimetric effects of anatomic changes.

### Statistical analysis

Descriptive statistics were calculated to characterize the dosimetric and volumetric changes of targets and OARs. Comparisons between replanning in the iCT with SOr-to-iCT and replanning in the rCT with SOrCT were analyzed using paired samples t test. All statistical analyses were conducted with SPSS 17.0 software (spss Inc., Chicago, IL). Differences were considered statistically significant when p < 0.05.

## Results

**Figure 2:**
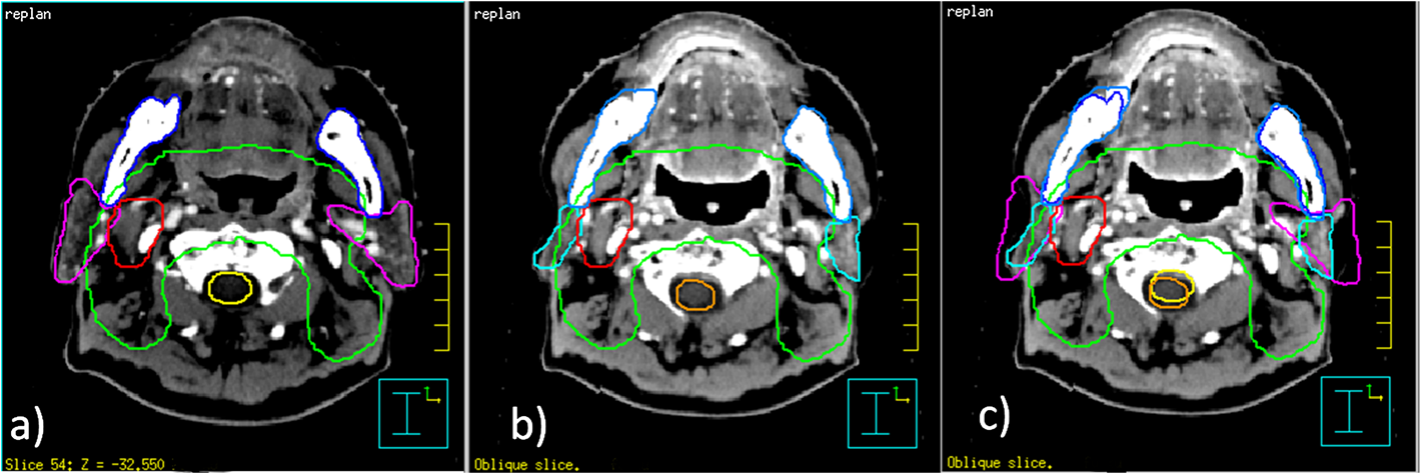
**Volumetric changes between iCT and rCT.** Volume changes between the initial planning CT and the rCT at the 23rd fraction; **a)** contours on the initial planning CT; **b)** contours on the rCT; **c)** contours on the initial planning CT projected on the rCT after image fusion.

Figure 2 shows typical volume changes of targets and OARs between the iCT and rCT at the 23rd fraction of one NPC patient, a) is the contours on the iCT; b) is the contours on the rCT; and c) is the contours on the iCT projected on the rCT after image fusion. The bony anatomy was well aligned after CT-CT image fusion. CTVs of all the NPC patients were still within physician’s expectation, therefore, no attempt had been tried to modify the contours of the CTVs. The shapes and locations of parotids were very different on the rCT compared to those on the iCT. Table 2 presents the quantitative volume changes of GTV and OARs. The average volume of GTV reduced from 78.83 ± 38.42 cm^3^ on the iCT to 71.44 ± 37.46 cm^3^ on the rCT, but with no significant difference (p = 0.42). Except for parotids, the volume of other OARs did not shrink very much at the 23rd fraction on the rCT. The average volume of the left parotid and right parotid were reduced from 19.91 ± 4.89 cm^3^ and 21.58 ± 6.16 cm^3^ on the iCT to 11.80 ± 2.79 cm^3^ and 13.29 ± 4.17 cm^3^ on the rCT (both p < 0.01), respectively.

**Table 2:** Volume changes between the initial planning CT (iCT) and the repeat CT (rCT) at 23rd fraction

**Unit(cm**^**3**^**)**	**Volumes in iCT**	**Volumes in rCT**	**Sig (p)**
GTV	78.83 ± 38.42	71.44 ± 37.46	0.42
Brainstem	28.77 ± 2.89	26.43 ± 3.03	0.22
Cord	25.52 ± 2.44	24.57 ± 2.30	0.44
Mandible	89.49 ± 14.33	89.23 ± 12.32	0.58
Left parotid	19.91 ± 4.89	11.80 ± 2.79	<0.01
Right Parotid	21.58 ± 6.16	13.29 ± 4.17	<0.01

**Figure 3:**
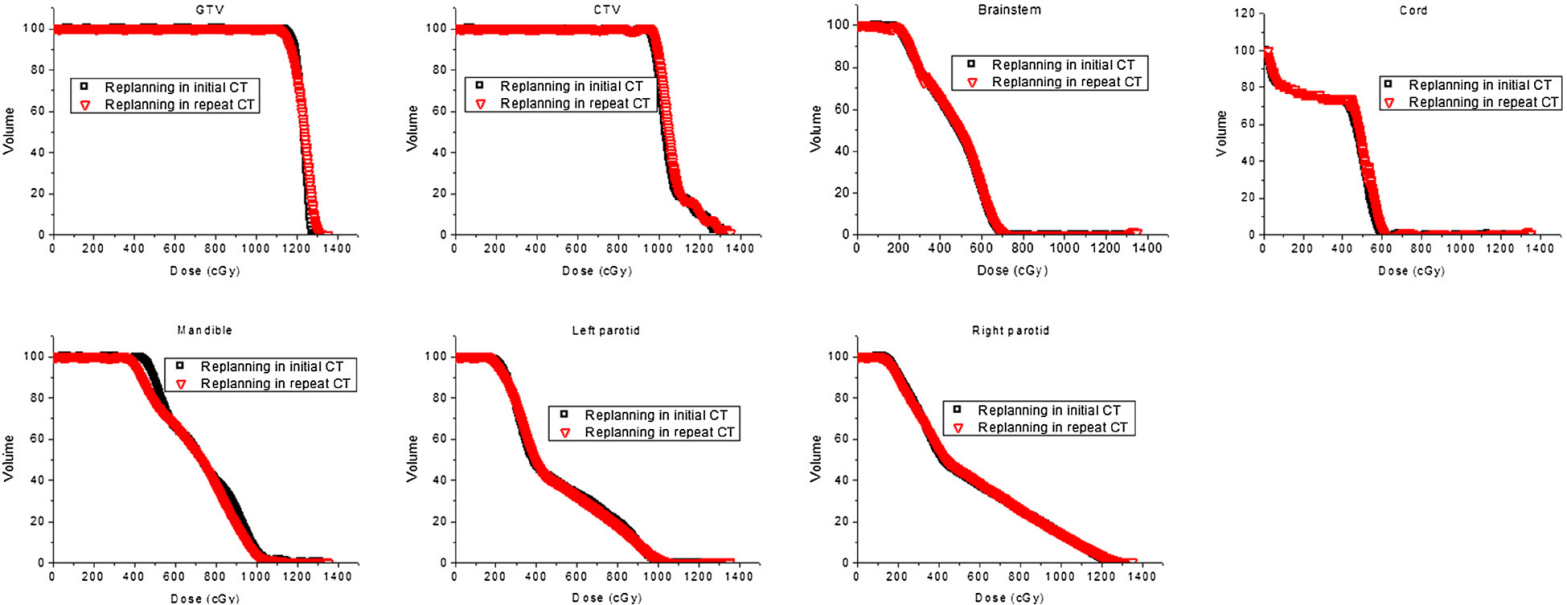
DVH comparisons between replanning based on the initial planning CT with propagated new contours from the rCT and replanning based on the rCT.

Dosimetric comparisons between replanning VMAT plans based on the iCT with SOr-to-iCT and replanning plans based on the rCT with SOrCT were shown in Figure 3 and Table 3. Figure 3 shows the DVH curves of targets and OARs of one NPC patient optimized in two CT sets with identical objective settings and parameters. The curves from two replanning VMAT plans were very close. Detailed average dosimetric characteristics were presented in Table 3. The V95 (percent volume covered by 95% prescription dose) of PTV_GTV_ for replanning in the iCT and in the rCT were 91.8 ± 7.2 and 93.0 ± 5.4 (p = 0.66), respectively. The V95 of PTV_CTV_ in these two replanning plans were 98.4 ± 1.3 and 98.7 ± 1.2 (p = 0.24), respectively. No significant dosimetric difference between replanning in the iCT and in the rCT was observed.

**Table 3:** Detailed dosimetric comparison between replanning based on the initial CT with propagated contours from the repeat CT and replanning based on the repeat CT

	**Initial CT**	**Repeat CT**	**Sig (p)**
PTV_GTV_ Dmax	1343.5 ± 65.9	1335.2 ± 29.7	0.66
PTV_GTV_ Dmean	1253.5 ± 40.3	1244.4 ± 13.7	0.42
PTV_GTV_ V93	96.9 ± 3.0	97.2 ± 2.6	0.79
PTV_GTV_ V95	91.8 ± 7.2	93.0 ± 5.4	0.66
PTV_CTV_ Dmax	1349.4 ± 61.3	1338.2 ± 29.0	0.55
PTV_CTV_ Dmean	1083.4 ± 31.5	1078.3 ± 23.7	0.67
PTV_CTV_ V93	98.9 ± 1.1	99.1 ± 0.9	0.12
PTV_CTV_ V95	98.4 ± 1.3	98.7 ± 1.2	0.24
Brainstem Dmax	859.7 ± 89.1	852.8 ± 89.7	0.5
Brainstem Dmean	457.2 ± 92.3	436.9 ± 110.5	0.15
Cord Dmax	692.0 ± 29.8	690.6 ± 31.3	0.9
Cord Dmean	413.3 ± 64.2	418.7 ± 63.5	0.17
Mandible Dmax	1146.9 ± 99.5	1131.6 ± 84.5	0.38
Mandible Dmean	691.7 ± 70.2	689.8 ± 60.5	0.24
Lt parotid Dmax	1134.0 ± 143.2	1139.1 ± 103.6	0.97
Lt parotid Dmean	519.4 ± 61.3	513.2 ± 57.2	0.17
Lt parotid V5	42.1 ± 8.5	41.8 ± 7.8	0.6
Rt parotid Dmax	1144.0 ± 95.8	1162.1 ± 107.3	0.21
Rt parotid Dmean	514.9 ± 32.8	511.8 ± 40.8	0.63
Rt parotid V5	39.5 ± 4.0	39.3 ± 4.8	0.82

Note: the unit for dose is cGy; V5 = the volume receiving 5 Gy.

**Figure 4:**
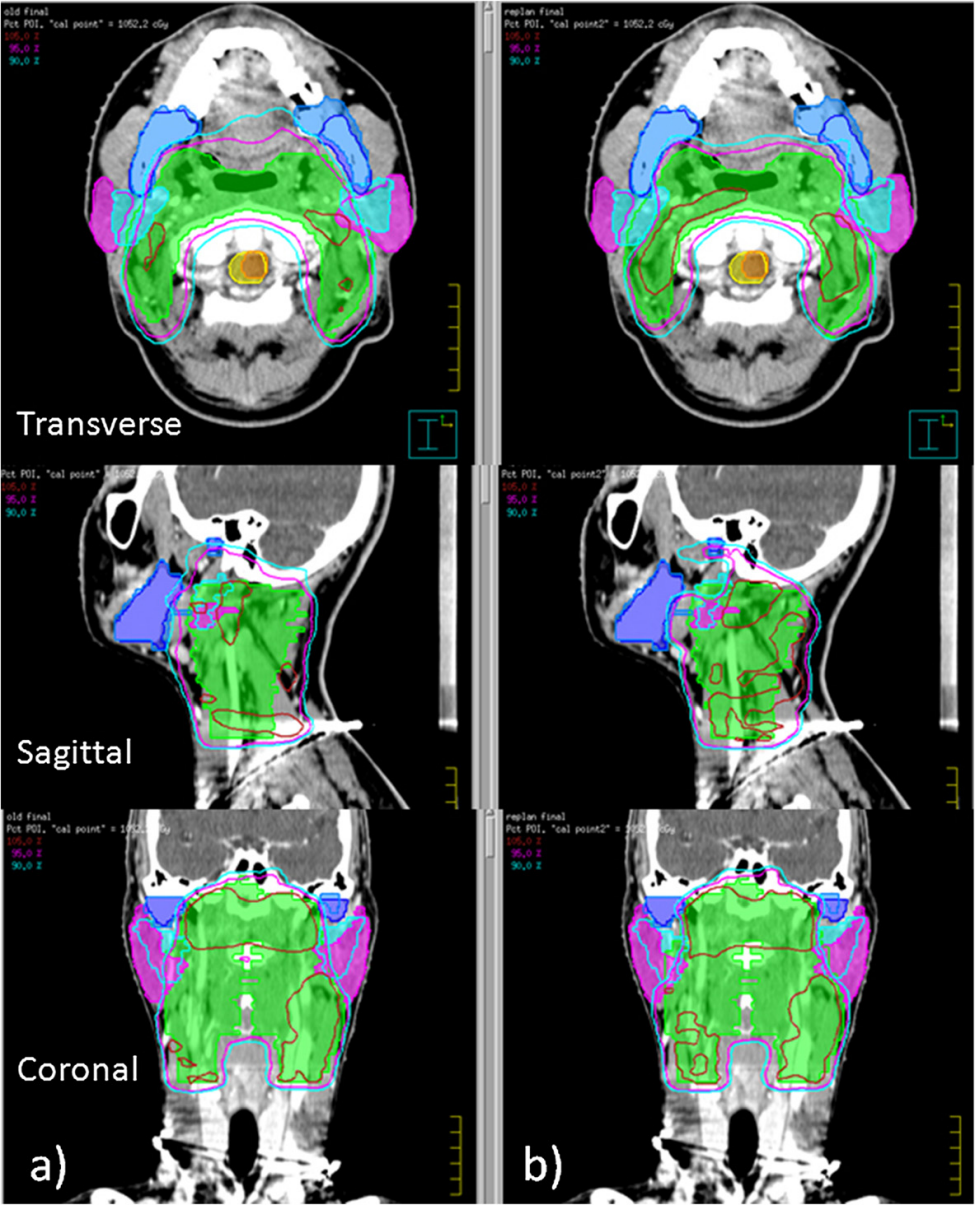
**Typical dose distributions.** A typical dose distribution of **(a)** the original VMAT plan delivered to the new contours projected in the initial planning CT, and **(b)** the re-optimized VMAT plan dose distribution based on the new contours projected in the iCT.

Figure 4 shows a typical dose distribution of (a) the original VMAT plan delivered to the new contours projected in the iCT, and (b) the re-optimized VMAT plan dose distribution based on the new contours projected in the iCT. The overdose delivered to the parotids due to anatomic changes was reduced by re-optimization. Detailed dosimetric changes due to anatomic change were summarized in Table 4. Compared to the initial plans, the average mean dose of the left and right parotid after re-optimization were decreased by 62.5 cGy (p = 0.05) and 67.3 cGy (p = 0.02), respectively, and the V5 (the volume receiving 5 Gy) of the left and right parotids were decreased by 7.8% (p = 0.01) and 11.2% (p = 0.001), respectively. No significant differences on the coverage of PTV_GTV_ and PTV_CTV_ and the dose delivered to other OARs were observed between the initial plans and re-optimized plans.

**Table 4:** Dosimetric comparisons of without replanning in the initial CT, replanning in the initial CT

	**Without replannng in iCT**	**Replanning in iCT**	**Sig (p)**
PTV_GTV_ Dmax	1332.6 ± 42.4	1343.5 ± 65.9	0.77
PTV_GTV_ Dmean	1257.2 ± 30.1	1253.5 ± 40.3	0.97
PTV_GTV_ V93	98.2 ± 1.7	96.9 ± 3.0	0.12
PTV_GTV_ V95	94.8 ± 4.7	91.8 ± 7.2	0.26
PTV_CTV_ Dmax	1341.8 ± 40.7	1349.4 ± 61.3	0.85
PTV_CTV_ Dmean	1087.2 ± 20.7	1083.4 ± 31.5	0.91
PTV_CTV_ V93	99.2 ± 0.7	98.9 ± 1.1	0.27
PTV_CTV_ V95	98.8 ± 0.9	98.4 ± 1.3	0.62
Brainstem Dmax	844.7 ± 95.4	859.7 ± 89.1	0.94
Brainstem Dmean	448.5 ± 76.4	457.2 ± 92.3	0.84
Cord Dmax	695.5 ± 44.2	692.0 ± 29.8	0.87
Cord Dmean	409.0 ± 54.7	413.3 ± 64.2	0.82
Mandible Dmax	1128.2 ± 95.0	1146.9 ± 99.5	0.76
Mandible Dmean	705.4 ± 70.9	691.7 ± 70.2	0.91
Lt parotid Dmax	1138.7 ± 114.6	1134.0 ± 143.2	0.99
Lt parotid Dmean	581.9 ± 87.1	519.4 ± 61.3	0.05
Lt parotid V5	49.9 ± 12.3	42.1 ± 8.5	0.01
Rt parotid Dmax	1151.8 ± 108.1	1144.0 ± 95.8	0.75
Rt parotid Dmean	581.2 ± 79.8	514.9 ± 32.8	0.02
Rt parotid V5	50.7 ± 8.3	39.5 ± 4.0	0.001

Note: the unit for dose is cGy, V5 = the volume receiving 5 Gy.

## Discussion

In this study, we evaluated the feasibility of a modified ART based on CT-CT image fusion. Our results indicated it’s feasible and accurate to based on CT-CT image fusion to treat NPC patients adaptively with replanning in the iCT with new contours projected from rCT. This study also demonstrated the importance of replanning for parotid protection during the VMAT treatment of NPC patients.

Based on CT-CT image fusion, DVHs and dosimetric characteristics of the replanning VMAT plans in the iCT with new contours projected from the rCT did not demonstrate significant difference compared to those of the replanning VMAT plans in the rCT directly. This results indicated instead of replanning in a different rCT set and transferring the original IMRT plan into the rCT, it is feasible to replan in the iCT and accumulate the dose based on CT-CT image fusion, using the rCT as a contour reference.

In this study, tremendous volume shrinkage of parotids were observed at the 23rd fraction after a dose of 46 Gy to PTV_CTV_ and 57.5 Gy to PTV_GTV_, respectively. The average shrinkage volumes of left parotid and right parotid were about 3.56 cm^3^ and 2.45 cm^3^, respectively. It was about average 24.9% and 15.2% of their initial volumes, respectively. This finding was similar to the reported results in previous studies. Barker et al. observed a median parotid volume loss of 28.1% at the end of treatment [14]. A largest average absolute volume shrinkage of 10 cm^3^ of parotids was observed at the 7th week for oropharyngeal cancer IMRT treatment in another study [15]. There were other different absolute and percentage volume changes of parotids were reported [16,17]. These differences might due to the differences in the prescription dose, the treatment fraction for the rCT, patient weight loss, and the prevalence of chemotherapy. No significant volume changes of other OARs was observed. There was also no significant volume changes for GTV between the iCT and the rCT (p = 0.66). Similar results were reported in the previous study for head-and-neck patients and NPC patients with rCTs [6,8].

Various dosimetric effects of position shift and anatomic changes on targets and OARs have been reported by different replanning studies. A dose decrease of 0.8-6.3 Gy and 0.2-7.4 Gy to 95% of PTV_GTV_ and PTV_CTV_ respectively in 92% of head-and-neck patients were reported by Hansen et al. [6]. Zhao et al. reported a dose decrease to CTV, but no dose decrease to GTV was observed for NPC patients [8]. Similar results were reported in the study of Wang et al. for NPC patients [7]. In our study, there was also no significant difference on the PTV_GTV_ coverage observed for new contours with and without re-optimization after CT-CT image fusion. In this study, the physicians decided not to change the CTV volume to ensure an adequate coverage for this high risk volume, so no dosimetric difference for CTV was reported.

No difference on the maximum dose of spinal cord was observed with and without replanning in our study, this was different from the reported results in previous two studies [6,8]. The dosimetric effects on brainstem and mandible was consistent with previous studies without significant effects [7,8]. It has been reported that the effect of volume changes of parotid glands is particularly important for patients with oro-and rhinopharynx tumors, in which the medial shift of the parotid corresponds to a shift toward the high-dose coverage region [15,18]. Similar dosimetric effects were demonstrated in our study. Replanning based on the new contours from rCT could decrease the mean dose the V5 for both parotids.

In this study, the patients with a rCT at the 23rd fraction were enrolled in the sake of data analysis consistence. Due to the difference in the prescription dose per fraction and the difference in the patient response, different time point for the rCT has been reported. Wang et al. suggested a necessary of rCT before 25th fraction for NPC patients [7]. Zhao et al. reported their rCT before 20th fraction [8]. Currently, clinician’s judgment plays the most important role in determining the need for a new CT scan based on the clinical observation. Future studies on identifying specific predictive factors for a rCT during radiotherapy will be helpful to realize the full benefits of adaptive radiotherapy.

## Conclusion

Patients with NPC undergoing dual arc VMAT had significant anatomic changes and dosimetric variations to parotids. It’s feasible to replanning in the iCT with contours propagated from a rCT based on CT-CT image fusion. It was accurate enough to identify the volume changes and to ensure safe dose to parotids with our modified adaptive radiotherapy scheme based on CT-CT image fusion for NPC patients. Future rCT and replanning study on other tumor sites will help to further verify the accuracy of CT-CT image fusion for this modified adaptive radiotherapy technique.

## Consent

Written informed consent was obtained from the patient for the publication of this report and any accompanying images.

## Competing interests

The authors declare that they have no competing interests.

## Authors’ contributions

Each author has participated sufficiently in the work to take public responsibility for appropriate portions of the content. XJ, CX designed the study. CH, YZ performed the study and analysis. JY, HY provided the patients’ images. The manuscript was written by XJ, all other authors helped and finally approved the final manuscript.

## References

[B1] Kam MK Chau RM Suen J Choi PH Teo PM Intensity-modulated radiotherapy in nasopharyngeal carcinoma: dosimetric advantage over conventional plans and feasibility of dose escalation Int J Radiat Oncol Biol Phys 2003 56 145 157 10.1016/S0360-3016(03)00075-0 12694833

[B2] Lee TF Ting HM Chao PJ Fang FM Dual arc volumetric-modulated arc radiotherapy (VMAT) of nasopharyngeal carcinomas: a simultaneous integrated boost treatment plan comparison with intensity-modulated radiotherapies and single arc VMAT Clin Oncol 2012 24 196 207 10.1016/j.clon.2011.06.006 21752615

[B3] Nutting CM Morden JP Harrington KJ Urbano TG Bhide SA Clark C Miles EA Miah AB Newbold K Tanay M Adab F Jefferies SJ Scrase C Yap BK A’Hern RP Sydenham MA Emson M Hall E PARSPORT trial management group: parotid-sparing intensity modulated versus conventional radiotherapy in head and neck cancer (PARSPORT): a phase 3 multicentre randomised controlled trial Lancet Oncol 2011 12 127 136 10.1016/S1470-2045(10)70290-4 21236730 PMC3033533

[B4] Han CH Chen YJ Liu AL Schultheiss TE Wong JY Actual dose variation of parotid glands and spinal cord for nasopharyngeal cancer patients during radiotherapy Int J Radiat Oncol Biol Phys 2008 70 1256 1262 10.1016/j.ijrobp.2007.10.067 18234431

[B5] Schwartz DL Current progress in adaptive radiation therapy for head and neck cancer Curr Oncol Rep 2012 14 139 147 10.1007/s11912-012-0221-4 22328127

[B6] Hansen EK Bucci MK Quivey JM Weinberg V Xia P Repeated CT imaging and replanning during the course of IMRT for head-and-neck cancer Int J Radiat Oncol Biol Phys 2006 64 355 362 10.1016/j.ijrobp.2005.07.957 16256277

[B7] Wang W Yang H Hu W Shan G Ding W Yu C Wang B Wang X Xu Q Clinical study of the necessity of replanning before the 25th fraction during the course of intensity-modulated radiotherapy for patients with nasopharyngeal carcinoma Int J Radiat Oncol Biol Phys 2010 77 617 621 10.1016/j.ijrobp.2009.08.036 20138444

[B8] Zhao L Wan Q Zhou Y Deng X Xie C Wu S The role of replanning in fractioned intensity modulated radiotherapy for nasopharyngeal carcinoma Radioth and Oncol 2011 98 23 27 10.1016/j.radonc.2010.10.009 21040992

[B9] Bhide SA Davies M Burke K McNair HA Hansen V Barbachano Y El-Hariry IA Newbold K Harrington KJ Nutting CM Weekly volume and dosimetric changes during chemoradiotherapy with intensity-modulated radiation therapy for head and neck cancer: a prospective observational study Int J Radiat Oncol Biol Phys 2010 76 1360 1368 10.1016/j.ijrobp.2009.04.005 20338474

[B10] Steenbakkers R Duppen J Fitton I Deurloo K Zijp L Eisbruch A Nowak P Van Herk M Rasch C Observer variation in delineation of nasopharyngeal carcinoma for radiotherapy: a 3-D analysis Int J Radiat Oncol Biol Phys 2004 60 S160 S161

[B11] Fung WWK Wu VWC Teo PML: Dosimetric evaluation of a three-phase adaptive radiotherapy for nasopharyngeal carcinoma using helical tomotherapy Med Dosim 2012 37 92 97 10.1016/j.meddos.2011.01.006 21945167

[B12] Wu SX Xie CY Jin XC Zhang P Simultaneous modulated accelerated radiation therapy in the treatment of nasopharyngeal cancer: a local center’s experience Int J Radiat Oncol Biol Phys 2006 66 s40 s46

[B13] Jin X Yi J Zhou Y Yan H Han C Xie C Comparison of whole field simultaneous integrated boost VMAT and IMRT in the treatment of nasopharyngeal cancer, Medical Dosimetry 2013 http://dx.doi.org/10.1016/j.meddos.2013.05.004 10.1016/j.meddos.2013.05.004 23973017

[B14] Barker JL Garden AS Ang KK O’Daniel JC Wang H Court LE Morrison WH Rosenthal DI Chao KS Tucker SL Mohan R Dong L Quantification of volumetric and geometric changes occurring during fractionated radiotherapy for head-and-neck cancer using an integrated CT/ linear accelerator system Int J Radiat Oncol Biol Phys 2004 59 960 970 10.1016/j.ijrobp.2003.12.024 15234029

[B15] Ricchetti F Wu B McNutt T Wong J Forastiere A Marur S Starmer H Sanguineti G Volume change of selected organs at risk during IMRT for oropharyngeal cancer Int J Radiat Oncol Biol Phys 2011 81 161 168 21306971 10.1016/j.ijrobp.2010.01.071

[B16] Lee C Langen KM Lu W Haimerl J Schnarr E Ruchala KJ Olivera GH Meeks SL Kupelian PA Shellenberger TD Mañon RR Evaluation of geometric changes of parotid glands during head and neck cancer radiotherapy using daily MVCT and automatic deformable registration Radiother Oncol 2008 89 81 88 10.1016/j.radonc.2008.07.006 18707786

[B17] Vasquez Osorio EM Hoogeman MS Al-Mamgani A Tequh DN Levendag PC Heijmen BJ Local anatomic changes in parotid and submandibular glands during radiotherapy for oropharynx cancer and correlation with dose, studied in detail with nonrigid registration Int J Radiat Oncol Biol Phys 2008 70 875 882 10.1016/j.ijrobp.2007.10.063 18262099

[B18] Schwartz DL Dong L Adaptive radiation therapy for head and neck cancer-can an old goal evolve into a new standard? J Oncol 2011 2011 1 14 10.1155/2011/690595 PMC2933914 20847944

